# Shared Decision-Making and Women’s Adherence to Breast and Cervical Cancer Screenings

**DOI:** 10.3390/ijerph15071509

**Published:** 2018-07-17

**Authors:** Jayoung Han, Paiboon Jungsuwadee, Olufunmilola Abraham, Dongwoo Ko

**Affiliations:** 1 Department of Pharmacy Practice, School of Pharmacy and Health Sciences, Fairleigh Dickinson University, 230 Park Ave, Florham Park, NJ 07932, USA; jayoung@fdu.edu; 2 Department of Pharmaceutical Sciences, School of Pharmacy and Health Sciences, Fairleigh Dickinson University, 230 Park Ave, Florham Park, NJ 07932, USA; paiboon@fdu.edu; 3 Social and Administrative Sciences Division, School of Pharmacy, University of Wisconsin-Madison, 777 Highland Ave, Madison, WI 53705, USA; olufunmilola.abraham@wisc.edu; 4 Department of Marketing, College of Business, Hankuk University of Foreign Studies, 107 Imun-ro, Imun 1-dong, Dongdaemun-gu, Seoul 02450, Korea

**Keywords:** cancer prevention, screening, women’s cancer, patient centered care, shared decision making

## Abstract

We examined the effect of shared decision-making (SDM) on women’s adherence to breast and cervical cancer screenings and estimated the prevalence and adherence rate of screenings. The study used a descriptive cross-sectional design using the 2017 Health Information National Trends Survey (HINTS) data collected by the National Cancer Institute. Adherence was defined based on the guidelines from the American Cancer Society and the composite measure of shared decision-making was constructed using three items in the data. Multivariable logistic regression was performed to examine the association between the SDM and adherence, controlling for cancer beliefs and socio-demographic variables. The analysis included 742 responses. Weighted to represent the U.S. population, 68.1% adhered to both breast and cervical cancer screening guidelines. The composite measure of SDM was reliable (α = 0.85), and a higher SDM score was associated with women’s screening adherence (b = 0.17; *p* = 0.009). There were still women who did not receive cancer screenings as recommended. The results suggest that the use of the SDM approach for healthcare professionals’ communication with patients can improve screening adherence.

## 1. Introduction

Breast cancer is the most diagnosed cancer among women, while cervical cancer is the leading cause of cancer deaths in women aged 20–39 in the United States [1]. The American Cancer Society (ACS) estimates that in 2018, 266,120 and 13,240 American women will be newly diagnosed with breast and cervical cancers, respectively [1]. The ACS also estimates that 40,920 and 4170 women will die of breast cancer and cervical cancer, respectively, in 2018 [1].

The probability of developing invasive breast cancer increases with age, while the probability of developing cervical cancer peaks at age 50 and younger [2]. Between 1990 and 2015, the age-adjusted mortality rate of breast cancer decreased by 39%, possibly because of a combination of early detection and advances in treatment of breast cancer [3]. Similarly, the incidence of cervical cancer is predicted to decrease by 2%, from 10,253 cases in 2010 to 10,041 cases in 2020 [4]. The reduction in cervical cancer is potentially influenced by the widespread use of the Papanicolaou (Pap) test, which has been demonstrated to have a strong association with the incidence of advanced stage cervical cancer and in reducing mortality [5,6]. Although the mortality rate of cervical cancer declined between 1975 and 2008, an increase was observed between 2008 and 2015 [1]. Also, cervical cancer death rates rose by 2.3% annually from 2011 to 2015 [4], implying that screenings may be warranted and should be promoted in high risk populations. 

Despite a potential for cancers to be diagnosed at an earlier stage, which has been shown to reduce cancer mortality rates [7], the cancer screening rates (e.g., mammography and Pap test) continue to be below the Healthy People 2020 targets issued by the U.S. Department of Health and Human Service [8]. Several factors have been found to be associated with an individual’s cancer screening decisions, including age, race/ethnicity, health insurance coverage, socioeconomic status, and education levels [3,9]. Also, it has been shown that women without the usual source of healthcare, such as a primary care physician, had a lower breast and cervical cancer screening rate [10]. To reduce such barriers, the Affordable Care Act (ACA) has included mammography and Pap test in mandated benefits, and the National Breast and Cervical Cancer Early Detection Programs have offered free screenings to the women without health insurance. Yet, the mammography received rate remains the same, while the Pap test screening rate has rather decreased since 2000. Thus, both rates are below the Healthy 2020 targets (mammography: 81.1%, Pap: 93%) [11].

To improve cancer screening rates, the U.S. Preventive Services Task Force (USPSTF) suggests that healthcare professionals use the shared decision-making (SDM) approach when recommending cancer screening to patients, especially for a “C” recommendation, in which the net benefit of screening is too small to be included in routine services, but can be given based on individual preference [12]. The principle of the SDM states that patients and clinicians work together and jointly make an informed healthcare decision [12]. The SDM has gained significant attention as a means for incorporating patient-centeredness into a healthcare decision, one of the six dimensions of healthcare performance proposed by the 2001 Institute of Medicine (IOM) report [13]. As noted by the USPSTF, the SDM (joint participation) is clearly differentiated from the informed consent (clinician disclosure) in terms of the degree of patient involvement and should satisfy both the “informed” and “joint” elements in the decision [12].

Furthermore, the SDM is particularly relevant to the cancer screening decision as various guidelines with different age cut-offs and recommendations [14] can confuse patients. For example, in 2002, the USPSTF recommended that women aged 40–49 years receive routine mammography every one to two years, which was not the case in their 2009 recommendation statement [15]. In contrast, the American Cancer Society (ACS) recommends a routine mammography every one to two years for women aged 40–44, but only recommends annual mammography for women aged 45–49 [16].

**Figure 1:**
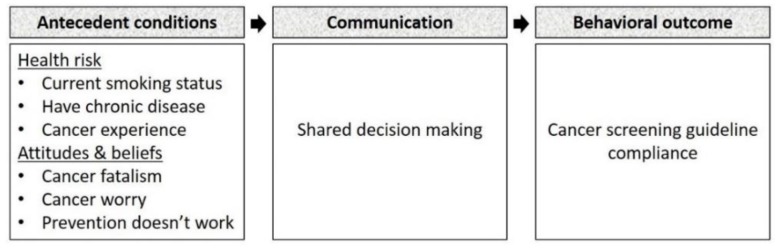
Conceptual framework.

Therefore, we aim to examine the association between SDM and women’s adherence to cancer screening guideline using the Transformation Model of Communication and Health Outcomes proposed by Kreps and colleagues [17] (Figure 1). The systems theory of input–process–output is the basis of the Kreps model in which the communication between the provider and the consumer (i.e., the process) transforms antecedent conditions (i.e., input) into health outcomes (i.e., output). The model suggests provider and consumer attributes and health risks as antecedent conditions, and classifies the health outcomes into cognitive, behavioral, and physiological outcomes. The model also states that the close coordination between communicators, including information sharing and relationship development, is essential in an effective transformation of inputs towards achieving the system goal [17]. This model has been used to frame the SDM in cancer patients [18,19].

Previous SDM studies have examined primarily cognitive outcomes such as patient satisfaction and knowledge [18]. In the context of cancer in women, a patient’s decision on receiving surgery (i.e., behavioral outcome) has been measured in breast cancer, and no studies have been done on cervical cancer patients [18]. Here, we focused on cancer screening adherence as a behavioral outcome of health communication using the SDM approach.

## 2. Materials and Methods

The study used the data from 2017 Health Information National Trends Survey (HINTS) 5 cycle 1 collected by the National Cancer Institute (NCI). This nationally representative mail survey targets non-institutionalized adults in the United States and has been administered since 2003 [20]. The HINTS 5 cycle 1 data were collected from January to May 2017 and released to the public in January 2018.

The population for this study were women aged 40–64 years at average risk. These women are recommended to receive mammogram and Pap tests regularly to prevent breast or cervical cancers based on the ACS guideline [16,21]. We excluded women at high risk, defined as having breast or cervical cancer history, because the screening guideline is set for women at average risk. We also excluded women who had not met a healthcare provider within the past 12 months, because we cannot measure their involvement in the shared decision-making process.

We defined women’s adherence to cancer screening as whether they received mammograms and Pap tests as recommended by the ACS. The 2015 ACS guideline for breast cancer screening for women at average risk recommends that women aged 45 to 54 years should receive annual screening and women aged 55 years and older should make the transition to biennial screening [16]. The 2012 ACS guideline for cervical cancer screening for women at average risk recommended that women aged 21–64 years should receive screening every three years [21].

Based on these guidelines, we created a binary variable to represent women’s adherence to breast cancer screening guideline. Women aged 45–54 years that received the most recent mammogram a year ago or less, and women aged 55–64 years that received it up to two years ago, were coded as 1 (adherence), and the other women aged 45–64 years were coded as 0 (non-adherence). All women aged 40–44 years were coded as 1 as the screening decision is made based on individual preference. We also created a binary variable to represent women’s adherence to cervical cancer screening guideline (women that received the most recent Pap test up to three years ago were coded as 1 and the other women were coded as 0). If both variables were equal to 1, we coded women’s adherence to cancer screening as 1; otherwise, 0.

The primary independent variable was the extent to which the patient and the healthcare professional communicated using the SDM approach. The SDM has been measured from the patient, clinician, and observer’s perspectives, of which the patient-reported SDM has been the majority [18]. We measured the SDM using the patient’s evaluation of their communication with all doctors, nurses, and other health professionals.

We used three four-point Likert scale questions (always–usually–sometimes–never), including “how often did they (healthcare professionals) do each of the following: involve you in decisions about your healthcare as much as you wanted, make sure you understood the things you need to do to take care of your health, and help you deal with feelings of uncertainty about your health or healthcare”. These questions asked if the patient felt invited to the healthcare decision and informed of needed healthcare information which captures both “joint” and “informed” elements of the SDM defined by the USPSTF [12]. All three items were reverse-coded to make higher numbers indicate more involved, informed, and helped with uncertainty (always = 4, usually = 3, sometimes = 2, never = 1). The composite measure of the SDM was constructed using these three items and Cronbach’s alpha, and item-total correlation coefficients were obtained to ensure the reliability of this measure.

We also measured beliefs about cancer as antecedent conditions of health communication. Previous studies found the associations of cancer screening with cancer worry and perceived risk [22]. We measured beliefs about cancer using three four-point Likert scale items (strongly agree (=1), somewhat agree (=2), somewhat disagree (=3), strongly disagree (=4)), asking the patient’s agreement to the following statements: “when I think about cancer, I automatically think about death”, “there’s not much you can do to lower your chances of getting cancer”, and “I would rather not know my chance of getting cancer”. We created binary variables representing beliefs about cancer (women who strongly agreed or agreed with the statement were coded as 1 and the other women were coded as 0).

We created several other binary variables to be included in the analyses, such as cancer experience (women with cancer other than breast or cervical cancers and/or women having family members with any type of cancer were coded as 1, and the other women were coded as 0), and chronic condition (women with at least one condition among diabetes, high blood pressure, heart condition, arthritis, lung disease, and depression were coded as 1 and the other women were coded as 0). Additionally, Hispanic ethnicity, insurance status, and smoking status were included as covariates in the analyses.

We created four categorical variables, including age (40–44, 45–49, 50–54, 55–59, 60–64), education (less than high school, high school graduate, some college, college graduate), race (white, black, others), and household income (below $20,000; $20,000 to $49,999; $50,000 to $99,999; $100,000 or more). Dummy variables to represent these four categorical variables were created for a logistic regression model. Reference values for age, education, race, and household income were age 60–64, less than high school, others, and $100,000 or more, respectively.

All estimates were weighted using personal weight and the jackknife replication method to incorporate the complex sampling process [23]. We obtained weighted and unweighted frequencies and percentages for binary and categorical variables using PROC SURVEYFREQ. We performed a series of bivariate analyses for adherence to screening guideline and used a Wald chi-square test for statistical significance. We performed the logistic regression, modeling adherence to the screening guideline using PROC SURVEYLOGISTIC. Observations with missing values were not included in the analyses. All analyses were performed in SAS 9.3 (SAS Institute, Cary, NC, USA).

## 3. Results

Among 3285 original responses, 742 responses were included in the analyses. Our samples represented the population that were white (76.4%), did not have Hispanic ethnicity (88.9%), and received more than high school education (73.7%). The majority of our samples had health insurance (94.9%). Nearly six out of ten women (58.9%) had been automatically thinking about death when they thought about cancer, and 40.6% of the women were afraid of finding out something wrong from the screening. About a quarter of the women (25.9%) thought that there was little to do to lower the chances of having cancer (Table 1).

**Figure 2:**
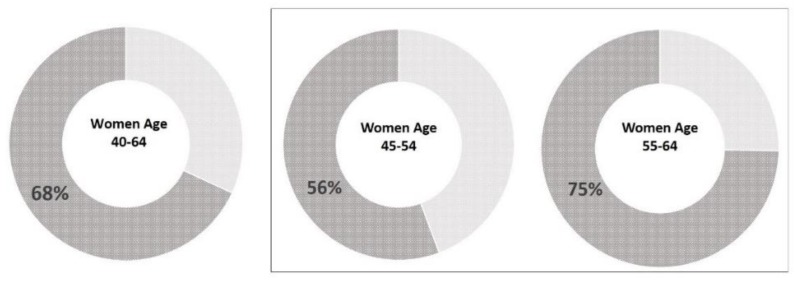
Estimated adherence rate for breast and cervical cancer screenings, United States, 2017. Note: Adherence rate was estimated based on American Cancer Society guideline published in 2015 (breast cancer) and in 2012 (cervical cancer). Charts in box represent the age groups that should receive both screenings. Breast cancer screening among the women aged 40–44 years were individual-based choices; therefore, not presented here. Age-specific estimates were calculated using the proportions of adherent women in each age group.

Weighted to represent the U.S. population, 68.1% of the women aged 40–64 years at average risk received regular screenings for both breast and cervical cancers as recommended. The estimated adherence rate largely differed between two age groups—women aged 45–54 years had a 19-point lower rate than the women aged 55–64 years (55.7% vs. 74.7%) (Figure 2). When considering cancer-type specifically, 75.5% as opposed to 86.5% of the women adhered to breast and cervical cancer screening guidelines, respectively.

Table 2 shows the estimated prevalence of mammogram and Pap tests among the women aged 40–64 years. Overall, about 5% and 1.5% of the women have never had mammogram or Pap test, respectively. It appears that a higher proportion of women aged 55–64 years than those aged 45–54 years received an annual mammogram and low- and middle-income women received less annual screenings.

The composite measure of SDM was reliable (Cronbach Alpha = 0.85) and the mean score was 9.99 (SD = 2.18) (Table 3). The logistic regression results indicated that a higher SDM score was associated with women’s adherence to cancer screening guidelines (b = 0.17; *p* = 0.009), after controlling for antecedent conditions (beliefs about cancer, health risks) and socio-demographic variables. Age groups of 45–49 years (b = −1.42; p = 0.001) and 50–54 years (b = −1.04; *p* = 0.043) were less likely to adhere to the guideline than the age group of 60–64 years (Table 4). Although cancer fatalism did not reach statistical significance at 0.05 level, its negative association with women’s adherence to the guideline is worth noting. The SDM might have relieved the patient’s fear of cancer.

## 4. Discussion

We found a positive association between the shared decision-making (SDM) approach and women’s adherence to breast and cervical cancer screening guidelines, after controlling for cancer beliefs and socio-demographic characteristics. We estimated women’s screening adherence rate using the most recent data collected after the release of the new breast cancer screening guideline in 2015. Three in ten women (31.9%) did not receive both screenings as recommended, and the non-adherence rate was particularly high among the women aged 45–54 years (44.3%).

Our study is among the first studies to measure both “informed” and “joint” dimensions of the SDM, as suggested by the USPSTF, between healthcare professionals and patients. Several clinical trials have used the SDM framework to explain the screening decisions on prostate cancer [24,25], colorectal cancer [26], and breast cancer [27], but they only focused on the information exchange aspect of the SDM by using the decision aids as an intervention (e.g., educational videotape). In other words, the studies actually measured the concept of informed decision-making instead of the SDM.

In addition, because of the lack of an agreed conceptual definition of the SDM, previous studies have used instruments like the control preference scale [28], measuring the “joint” dimension of the SDM, or used a single invalidated item [29,30,31,32] or used the items capturing only the “informed” aspect of the SDM [33]. These approaches commonly failed to capture both “informed” and “joint” dimensions of the SDM, which might have resulted in mixed reports concerning the relations between SDM and the treatment decisions. Some found no association between the SDM and breast surgery decision [30,32,33], while others found a positive association with surgery decision [29] or the receipt of guideline concordant depression care [31]. Furthermore, the studies using these scales specifically focused on the patient’s communication with the physician, while our study addressed the patient’s interaction with any of the healthcare professionals, and is thus closer to the real world decision environment and provides broader implications.

Another strength of our SDM measurement is that we accounted for whether the patient’s feeling was addressed in the communication, which differentiates SDM from the informed decision model. The USPSTF stressed the need for helping the patient’s feeling of uncertainties around a screening decision. A patient’s screening decision is known to be influenced by not only cognitive factors (e.g., processing risk information), but also affective factors (e.g., emotional responses to risk information) [34]. Slovic and colleagues argued that the patient may not use numerical risk information in decision “unless it makes an affective connection” [35]. In other words, the patient may not properly weigh risks and benefits of screening and make informed decision if their feelings are not handled properly. Furthermore, when facing a decision relating to a dreaded disease, affective factors increase perceived risk of the disease, which then drive an individual’s preventive action [22]. A previous study showed that women with a higher perceived risk of breast cancer were more likely to get a mammogram [36]. In our study, 57% of the women strongly agreed or agreed that they automatically think about death when thinking about cancer, indicating that women do perceive cancer as a dreaded disease. Therefore, helping patients handle their feelings would improve screening decisions, which should be part of the SDM measurement.

We defined adherence as having received the screenings within the recommended interval, as done by previous screening adherence studies [37,38]. Under this definition, annual mammography among women aged 55 years or older, or annual/biennial Pap tests were all considered as being adherent to screening guidelines. Some studies defined shorter-interval screening as over screening and distinguished it from guideline-adherent screening [39,40]. However, this approach should be taken with caution because it is too strict to reflect the real world practice, where it takes time for healthcare professionals to become aware and endorse the new guideline [41]. This approach could substantially underestimate the screening adherence rate, considering that significant changes were made in breast cancer guidelines only two years ago. Such inconsistency in screening adherence measurement was also acknowledged in a recent systematic review on cancer screening adherence [40].

Our age-specific prevalence estimates of mammography and Pap tests were generally higher than the existing statistics. Sixty-percent of women aged 45–54 received annual mammography (vs. 54% in ACS report) and 52% of those aged 40–44 (vs. 38% in ACS report) [42]. In addition, 85.2% of women aged 40–64 received a triennial Pap test (vs. 79.9% in CDC report) [11]. This is probably because we included women who had met a healthcare professional at least once within the past 12 months. Patients with a recent health appointment are likely to have a usual care source and have more chances to get screening recommendations from the physician, both of which are strong factors related to screening adherence [40,43]. Also, patients with a recent health visit may be more health conscious and respond better to health promotion strategies.

It is noteworthy that the screening rate barely reached the Healthy 2020 target even among the women with a recent health encounter. We can only make general comparisons because the age range studied was different from the Healthy2020, but it is certainly informative. The Healthy 2020 aims to reach the goals that 81.1% of the women aged 50–74 and 93% of the women aged 21–65 receive biennial mammography and triennial Pap tests [44], respectively. We estimated that 87.3% of the women aged 50–64 received a mammography within the two years and 85.2% of those aged 40–64 received a Pap test within three years.

Women aged 45–54 years were significantly less likely to adhere to both guidelines compared with the women aged 60–64 years, after controlling for socio-demographic variables. This age range could cause significant confusion to patients and clinicians because of disagreement among the breast cancer screening guidelines from different sources, which might have resulted in the low non-adherence rate (cervical cancer screening guidelines are concordant). The USPSTF recommended yearly or biennial screening among the women older than 40 years in its 2002 guidelines and changed them to recommend biennial screening among women aged 50–74 in its 2014 guidelines [45]. The American Academy of Family Physicians (AAFP) and the International Agency for Research on Cancer (IARC) agree with the 2014 USPSTF, but the American College of Obstetricians and Gynecologists (ACOG) and the American College of Radiology (ACR) recommend that women aged 40 to 74 with average risk get a mammogram every year [14]. As patients usually discuss preventive services, including cancer screening, with their primary care physicians, they might have gotten different screening recommendations depending on which guideline their PCP endorsed.

There are several limitations of this study. First, we could not exclude from our sample the BRCA1/BRCA2 mutation carriers, who are defined as high-risk individuals by the ACS [16], for want of data to identify individual’s genetic mutations. However, these mutations rarely happen in the general population [42], so the impact on our results were minimal. Also, women with Hispanic ethnicity and low education attainment were underrepresented in our sample. Hispanics are known to be less adherent to the treatment, particularly because of language barriers [42], so our adherence rate could have been overestimated.

In addition, because a mammography and a Pap test are mandatory preventive care benefits under the ACA, we assumed that all insured patients had the same coverage for these services. However, insurers might have detailed the benefits, including screening interval, based on different guideline sources. For example, the ACA endorsed the 2002 USPSTF guideline for breast cancer screening, so public insurances and marketplace plans still cover an annual mammography for women aged 40 or older. If there were private plans that adopted the guideline recommending against an annual mammography, which we could not identify this from the data, the screening prevalence might have been impacted by this variation. 

Similarly, we did not have the variable representing which guideline was endorsed by the healthcare professional that communicated with the patient. A recent nationwide study showed that physicians trust breast cancer screening guidelines from various sources—26% trust ACOG; 23.8% trust ACS; and 22.9% trust USPSTF guidelines—and make corresponding recommendations [46]. Although physician recommendation has been associated with the patient’s screening adherence [47], the variation in physician’s guideline endorsement is not of much concern in our study because we did not take screening interval into account in our adherence measure, which is the component discordant among the guidelines.

Lastly, we controlled for the patient-related factors as antecedents of the relationship between the SDM and adherence based on the adapted TMCHO model, but did not adjust for provider-related factors in our analyses. Medical specialty, physician experience, or communication training could have influenced our results. Also, all measures were constructed based on self-reported data.

## 5. Conclusions

Women’s adherence to breast and cervical cancer screening guidelines needs further improvement to meet the Healthy 2020 target rate. Shared decision-making (SDM) was found to be associated with women’s screening adherence, suggesting its potential role in early detection and treatment of cancer in women. 

Shared decision-making (SDM) appears to be a gateway for success in improving patients’ adherence to cancer screening recommendations. Physician communication, mainly task-oriented, has been shown to enhance patients’ adherence, which largely varies across disease conditions and types of medical activities [43]. Our results suggest that having the patient feel as an equal communication partner can improve the patient’s screening adherence, even though the communication context is not specifically tailored to the screening. In addition, the SDM effect is not limited to the physician; it can apply to any healthcare professional who interacts with patients. Community pharmacists are one example; their discussions about screening opportunities with patients and assessment of risk were found to improve the cancer screening rate [48]. With SDM training, community pharmacists could further expand their preventative role.

## Figures and Tables

**Table 1:** Sample characteristics (*n* = 742).

Characteristic	Unweighted		Weighted	
	*N*	%	*N*	%
Age, years				
40–44	102	13.7	7,293,240	15.4
45–49	136	18.3	12,628,813	26.6
50–54	132	17.8	11,329,835	23.9
55–59	168	22.6	8,143,501	17.2
60–64	204	27.5	8,035,632	16.9
Race				
White	500	70.2	34,947,178	76.4
Black	143	20.1	7,029,279	15.4
Others	69	9.7	3,787,550	8.3
Ethnicity				
Hispanic	79	11.1	656,486	11.1
Non-Hispanic	632	88.9	1,948,718	88.9
Education				
<High school	26	3.5	1,661,447	3.5
High school diploma	138	18.7	1,0793,958	22.9
Some college	232	31.5	16,558,808	35.1
College diploma	341	46.3	18,218,659	38.6
Income, $				
<20,000	133	19.5	7,616,523	17.4
20,000–50,000	154	22.6	9,430,340	21.5
50,000–100,000	202	29.6	12,890,436	29.4
100,000+	193	28.3	13,852,838	31.6
Health insurance				
Yes	706	95.3	44,796,480	94.9
No	35	4.7	2,412,043	5.1
Have chronic diseases				
Yes	521	70.9	31,448,143	66.8
No	214	29.1	15,607,023	33.2
Have cancer experience				
Yes	68	9.2	34,663,300	24.1
No	673	90.8	11,008,756	75.9
Current smoker				
Yes	118	15.9	7,321,669	15.4
No	623	84.1	40,097,441	84.6

**Table 2:** Prevalence estimates of mammography and Pap test (%), Women 40–64, United States, 2017.

	Mammography				Pap Test		
Characteristic	Never Had	Within the Past Year	1–2 Years Ago	2+ Years Ago	Never Had	Within the Past 3 Years	3+ Years Ago
**Overall**	4.9	63.8	19.6	11.7	1.4	85.2	13.4
**Age, years**							
40–44	14.7	52.0	19.6	13.7	2.9	91.2	5.9
45–49	9.1	52.3	26.5	12.1	3.8	85.0	11.2
50–54	1.5	67.9	21.4	9.2	0.0	90.9	9.1
55–59	1.8	71.5	15.8	10.9	0.6	86.1	13.3
60–64	2.0	68.1	17.2	12.7	0.5	77.8	21.7
**Race**							
White	4.0	64.1	19.1	12.9	0.4	86.3	13.3
Black	6.5	66.2	20.1	7.2	0.7	88.6	10.7
Others	7.4	60.3	22.1	10.3	7.3	72.5	20.2
**Ethnicity**							
Hispanic	6.6	67.1	17.1	9.2	4.0	82.9	13.1
Non-Hispanic	4.5	63.9	19.9	11.8	1.1	85.7	13.2
**Education**							
<HS	8.7	60.9	21.7	8.7	4.2	79.2	16.6
HS diploma	6.6	60.3	19.9	19.9	2.2	84.7	13.1
Some college	7.0	61.6	19.2	12.2	1.7	83.1	15.2
College diploma	2.1	66.9	19.9	11.1	0.3	87.3	12.4
**Income, $**							
<20,000	9.3	55.8	22.5	12.4	4.5	77.4	18.1
20,000–50,000	7.8	53.9	24.0	14.3	1.3	77.1	21.6
50,000–100,000	1.5	70.9	16.1	11.6	0.5	88.3	11.2
100,000+	3.6	65.8	20.7	9.8	0.0	91.2	8.8

Note. HS stands for high school. Prevalence was estimated based on when the patient had the most recent screening and weighted to represent the U.S. population.

**Table 3:** Shared decision making and beliefs about cancer items, %.

**Items**	***N***	**Always**	**Usually**	**Sometimes**	**Never**	**M**	**SD**	**α**	**Item**
**Shared decision making**									
Involve you in decision about your healthcare as much as you wanted	735	54.1	29.1	14.1	2.6	1.65	0.82	0.77	0.75
Make sure you understood the things you need to do to take care of your health	740	61.1	28.5	9.1	1.4	1.50	0.71	0.78	0.74
Help you deal with feelings of uncertainty about your health or healthcare	737	45.8	30.4	16.3	7.5	1.85	0.95	0.83	0.68
Test scale								0.85	0.72
**Items**	***N***	**Strongly Agree**	**Agree**	**Disagree**	**Strongly Disagree**		**M**	**SD**	
**Beliefs about cancer**									
When I think about cancer, I automatically think about death	729	22.2	35.1	28.3	14.4		2.35	0.98	
I would rather not know my chance of getting cancer	735	11.2	24.9	27.6	36.3		2.89	1.02	
There’s not much you can do to lower your chances of getting cancer	729	5.9	18.9	41.7	33.5		3.04	0.86	

**Table 4:** Summary of logistic regression analysis for variables predicting women’s adherence to cancer screening guideline by shared decision-making, controlling for beliefs about cancer and socio-demographic variables.

Parameter	B	SE	*p*-Value	Odds
Shared decision making	0.17	0.06	0.009	1.19
Beliefs about cancer				
When I think about cancer, I automatically think about death	−0.26	0.14	0.063	0.77
I would rather not know my chance of getting cancer	0.11	0.16	0.503	1.11
There is not much you can do to lower your chances of getting cancer	0.10	0.17	0.564	1.11
Age, years				
40–44	0.72	0.86	0.407	2.05
45–49	−1.42	0.40	0.001	0.24
50–54	−1.04	0.50	0.043	0.35
55–59	−0.10	0.46	0.824	0.90
Race				
White	0.09	0.20	0.658	1.10
Black	0.14	0.21	0.515	1.15
Hispanic	0.43	0.56	0.441	1.54
Education				
High school diploma	−1.57	0.91	0.089	0.21
Some college	−1.24	0.92	0.186	0.29
College diploma	−1.23	0.96	0.203	0.29
Income, $				
<20,000	−0.86	0.61	0.165	0.42
20,000–50,000	−0.31	0.57	0.594	0.74
50,000–100,000	−0.14	0.45	0.752	0.87
Health insurance	0.47	0.55	0.389	1.61
Have chronic diseases	−0.41	0.43	0.344	0.66
Have cancer experience	0.02	0.34	0.953	1.02
Current smoker	−0.36	0.45	0.423	0.70
Constant	0.99	1.55	0.528	
Model χ^2^(*df*)	584.19 (11.9), *p* < 0.001			
N	599			
Pseudo R^2^	0.17			

Note. Reference categories are 60–64 years old (age), others (race), less than high school (education), and $100,000+ (income). Shared decision-making score ranged from 7 to 28, high score indicating more engagement in shared decision-making approach. Three items measuring beliefs about cancer scaled from 1 for strongly agree to 4 for strongly disagree.
